# Comparing Electrochemical Passivation and Surface Film Chemistry of 654SMO Stainless Steel and C276 Alloy in Simulated Flue Gas Desulfurization Condensates

**DOI:** 10.3390/ma17081827

**Published:** 2024-04-16

**Authors:** Luhai Liao, Yifan Cheng, He Zhang, Xuwen Yuan, Fengguang Li

**Affiliations:** 1 Key Laboratory for Ferrous Metallurgy and Resources Utilization of Ministry of Education, Wuhan University of Science and Technology, Wuhan 430081, China; llh_ustb123@163.com; 2 Hubei Provincial Key Laboratory for New Processes of Ironmaking and Steel Making, Wuhan University of Science and Technology, Wuhan 430081, China; 3 School of Materials Science and Engineering, Hubei University of Automotive Technology, Shiyan 442002, China; 202211148@huat.edu.cn (Y.C.); 18752915197@163.com (H.Z.); 20210015@huat.edu.cn (X.Y.)

**Keywords:** super austenitic stainless steel, nickel-based alloy, electrochemical passivation behavior, flue gas desulfurization, passive film

## Abstract

This research examines the behavior of electrochemical passivation and the chemistry of surface films on 654SMO super austenitic stainless steel and C276 nickel-based alloy in simulated condensates from flue gas desulfurization in power plant chimneys. The findings indicate that the resistance to polarization of the protective film on both materials initially rises and then falls with either time spent in the solution or the potential of anodic polarization. Comparatively, 654SMO exhibits greater polarization resistance than C276, indicating its potential suitability as a chimney lining material. Mott–Schottky analysis demonstrates that the density of donors in the passive film formed on 654SMO exceeds that on C276, potentially due to the abundance of Fe oxide in the passive film, which exhibits the characteristics of an n-type semiconductor. The primary components of the passive films on both materials are Fe oxides and Cr oxides. The formation of a thin passive film on C276 in the simulated condensates is a result of the low Gibbs free energy of nickel oxide and low Cr content. The slower diffusion coefficient of point defects leads to the development of a thicker and more compact passive film on the surface of 654SMO.

## 1. Introduction

Flue gas of the thermal power plant enters the chimney after flue gas desulfurization (FGD). When it meets with the outside cold air, the flue gas with high humidity easily condenses, and the small acid droplets formed flow into the chimney, which seriously corrode the chimney lining materials [1,2,3,4,5,6]. These acid droplets are mainly composed of sulfuric acid and contain a certain amount of nitric acid and hydrochloric acid. The mixed acid seriously aggravates the corrosion damage of the chimney lining [7,8,9]. Therefore, the corrosion behavior of chimney lining materials cannot be ignored.

Pure titanium, titanium-based alloy, and nickel-based alloy are often used in the FGD system [10,11,12,13]. However, the cost of these materials is high. In recent years, high-alloyed super austenitic stainless steels and super duplex stainless steels have been proposed as chimney lining materials to resist the severe corrosion. Dou et al. [14,15] investigated the corrosion behavior of 254SMO super austenitic stainless steel in the FGD system, and they concluded that 254SMO exhibited excellent corrosion resistance in the flue gas condensates. Cui et al. [16,17] studied the effect of temperature, dissolved oxygen concentration, and the pH of solution on the surface electrochemical behavior of 2507 super duplex stainless steel in simulated flue gas desulfurization condensates. They found that 2507 stainless steel showed good corrosion resistance in condensates at different temperature and pH values. Zheng et al. [18] compared the corrosion resistance of C22 nickel-based alloy, TC4 titanium alloy, and Q235 carbon steel with surface laser cladding C22 alloy in FGD condensates. They found that compared with C22 alloy, C22 coating also showed good corrosion resistance, but it was more sensitive to local corrosion, and TC4 alloy exhibited poor corrosion resistance. Wang et al. [19] reported that the corrosion resistance of 316L and C276 alloy in simulated condensate was inferior to that of 310S and 254SMO. Zeng et al. [20] believed that martensitic stainless steel, austenitic stainless steel, and duplex stainless steel are good candidates for the flue gas desulfurization system of high temperature boilers, while nickel-based alloys such as C22 and C276 are more suitable for the flue gas system of biomass boilers and solid waste boilers.

The results of the above published literature indicate that highly alloyed austenitic stainless steels and duplex stainless steels may exhibit superior corrosion resistance in an FGD system compared to nickel-based alloys. However, the reason why super stainless steels show excellent corrosion resistance in an FGD system is still worth further investigation. This study investigates the passivation behavior of 654SMO super austenitic stainless steel and C276 nickel-based alloy in simulated desulfurization flue gas condensates through electrochemical analysis. X-ray photoelectron spectroscopy (XPS) was utilized to assess the composition and thickness of the passive film on the 654SMO stainless steel and C276 alloy.

## 2. Experimental

### 2.1. Materials and Solutions

654SMO super austenitic stainless steel and C276 nickel-based alloy sheets, which were 3 mm thick, were utilized in the experiment. Table 1 displays the chemical compositions and PREN value of both materials. Comparing C276 to 654SMO from Table 1, C276 has higher Mo and Ni content but lower Cr content. Despite C276 having a higher PREN value than 654SMO, it does not necessarily mean that C276 is more corrosion-resistant than 654SMO in FGD environments.

The simulated flue gas condensate composition can be found in Table 2 [19]. The temperature of the condensate typically falls within the 40~80 °C range. This study investigates the electrochemical properties of the two materials in simulated condensate at 80 °C.

### 2.2. Electrochemical Measurements

The sheets utilized for conducting electrochemical tests were cut to dimensions of 10 × 10 × 3 mm. Subsequently, they were wet-ground with 3000 grit SiC paper, followed by polishing with 0.5 μm alumina polishing powder. The sheets were then rinsed with distilled water and air-dried. For the electrochemical analysis, a standard three-electrode cell was employed. The cell consisted of a platinum sheet as the counter electrode and a silver/silver chloride (Ag/AgCl) saturated potassium chloride as the reference electrode.

Prior to testing, the working electrode was potentiostatically at −1.0 V_Ag/AgCl_ for 5 min to remove the air-formed oxides. Potentiodynamic polarization curves were performed from 0.4 to 1.1 V_Ag/AgCl_ with a scan rate of 0.5 mV/s. Open circuit potentials of the samples were measured at different immersion time (from 3 h to 96 h). Potentiostatic polarization was performed at 0.1, 0.2, 0.3, 0.4, 0.5, 0.6, and 0.7 V_Ag/AgCl_ for 1 h. Electrochemical impedance spectroscopy (EIS) tests were carried out for samples after immersing in solution for 3 h to 96 h and for samples after potentiostatic polarization for 1 h at 0.1 V_Ag/AgCl_ to 0.7 V_Ag/AgCl_. EIS experiments were conducted across a frequency range of 100 kHz to 10 mHz using a sinusoidal voltage of 10 mV amplitude. The collected EIS data were then processed and analyzed through ZsimpWin 3.3 software. Mott–Schottky tests were carried out at a constant frequency of 1 kHz, sweeping from −0.4 V to 0.8 V at a rate of 25 mV with a 10 mV signal amplitude. To ensure the reliability of the results, each electrochemical test was repeated three to five times for reproducibility assessment.

### 2.3. Characterization of Passive Film

XPS analysis was conducted on the specimens following exposure to simulated condensate at 80 °C for 24 h employing the Thermo Scientific ESACLAB Xi+ XPS analyzer (Thermo Scientific, Waltham, MA, USA). The specimens were sputtered post-immersion through Ar+ ion bombardment every 10 s using a 1 kV ion beam for depth profile acquisition. The XPS data were analyzed utilizing the Avantage 6.7 software.

## 3. Results and Discussion

### 3.1. Electrochemical Behavior at Different Immersion Times

#### 3.1.1. Open Circuit Potential and Potentiodynamic Polarization

**Figure 1:**
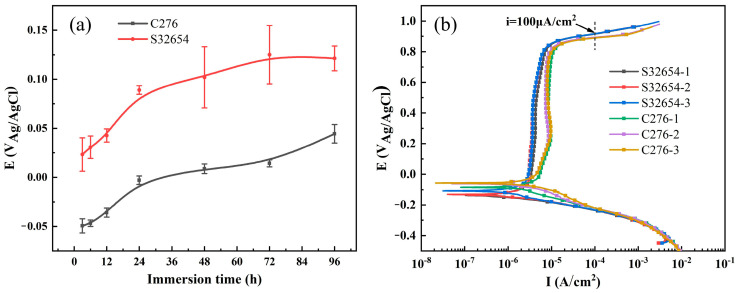
Open circuit potential as a function of immersion time (**a**) and the potentiodynamic polarization curves of the two materials in the simulated flue gas condensate at 80 °C (**b**).

The variation in OCP of the two materials with immersion time and the potentiodynamic polarization curves are shown in Figure 1a,b. It can be seen that the OCP increases continuously with the immersion time gradually reaching a stable one. Compared with C276 alloy, the higher open circuit potential of 654SMO indicates a lower corrosion tendency in FGD condensate [21]. In addition, no obvious corrosion pits were detected on the surfaces of the two materials after 96 h immersion.

There are obvious passivation regions in the polarization curves for the two materials, and activation dissolution peak is not observed in Figure 1b, which indicates that the oxide film formed in the testing process causes the passivation of the materials [22]. The potential when current density reaches 100 μA/cm^2^ is selected as the transpassive potential (E_tr_). The passivation current density (i_p_) is obtained from the average value of the current density in the passive region. Table 3 gives the electrochemical parameters obtained from potentiodynamic polarization curves. A lower i_p_ value and a higher E_tr_ value of 654SMO stainless steel suggests that the dissolution rate of passive film of 654SMO is lower than that of C276, and high-alloyed 654SMO is more protective in FGD condensate.

#### 3.1.2. Electrochemical Impedance Spectroscopy

**Figure 2:**
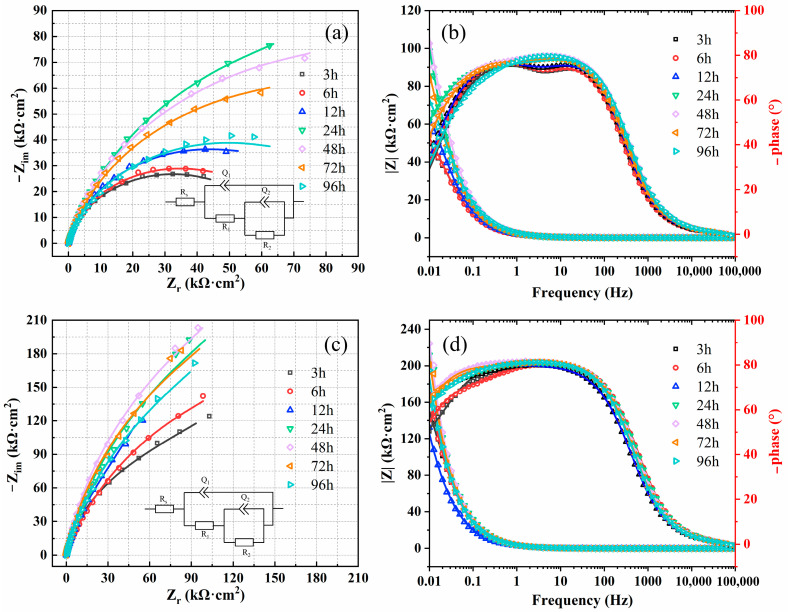
Nyquist (**a**,**c**) and Bode (**b**,**d**) diagrams of C276 alloy (**a**,**b**) and 654SMO stainless steel (**c**,**d**) at different immersion time.

EIS tests were performed on the samples of the two materials immersed in simulated solution at 80 °C for 3 h to 96 h. The results of EIS are shown in Figure 2 in the form of Nyquist and Bode plots, respectively. It can be seen that all curves of the two materials show an incomplete semi-circular arc. Initially, the diameter of the arc of capacitance increases before subsequently decreasing as the immersion time extends from 3 h to 96 h. In contrast to the expected capacitance behavior, the phase angle of each curve displayed on the Bode plot is consistently below 90°, an outcome attributed to the unevenness of the electrode surface. Hence, the substitution of an ideal capacitor with a constant phase element (*CPE*) becomes imperative to accurately represent the equivalent circuit. The impedance of the *CPE* is usually expressed as follows [23]:

(1) $$Z_{CPE}=Q^{-1}\cdot {\left(j\cdot \omega \right)}^{-n}$$

where *Q* is the *CPE* capacitance, *ω* is the angular frequency (rad/s), *j* is the imaginary number unit (*j*^2^ = −1), *n* is the *CPE* adjustment parameter, when *n* = 1, *CPE* shows the characteristics of an ideal capacitor, when *n* = 0.5, *CPE* shows a semi-infinite diffusion impedance, for non-ideal capacitor, *n* is generally 0.5~1.

The data presented in Table 4 outline the key parameters of each element determined through fitting with an equivalent circuit. In Table 4, the solution resistance (R_s_), passivation film resistance (R_1_), charge transfer resistance (R_2_), oxide film capacitance (Q_1_), double layer capacitance (Q_2_), ChPE exponent for oxide film (n_1_), and *CPE* exponent for double layer (n_2_) are detailed. It is observed that the total polarization resistance (R_p_ = R_1_ + R_2_) of the materials increases first and then decreases with prolongation of immersion time, in alignment with findings from Nyquist plots. Notably, the polarization resistance of 654SMO stainless steel surpasses that of C276 alloy, indicating that the passive film formed on 654SMO in simulated FGD condensate is more stable and offers superior corrosion protection.

#### 3.1.3. Mott–Schottky Analysis

Mott–Schottky (M–S) tests are widely used to investigate the semiconductor properties of the passive film formed on the metal surface. In the Mott–Schottky theory, the relationship between the space layer charge capacitance and the applied potential of n-type and p-type semiconductors can be expressed by the following relationship [24,25]:

(2) $$C^{-2}=\frac{2}{\varepsilon \varepsilon_{0}eN_{D}}\left(E-E_{FB}-\frac{KT}{e}\right)\;\text{n-type}\;$$

(3) $$C^{-2}=-\frac{2}{\varepsilon \varepsilon_{0}eN_{A}}\left(E-E_{FB}-\frac{KT}{e}\right)\;\text{p-type}$$

where the semiconductor capacitance is denoted by *C*, the applied potential by *E*, the dielectric constant by *ε*, the vacuum dielectric constant by *ε*_0_ (8.854 × 10^−14^ F/cm), the flat band potential by *E_FB_*, the Boltzaman constant by *K* (1.3 × 10^−23^ J/K), and the donor density and acceptor density by *N_D_* and *N_A_*, respectively.

**Figure 3:**
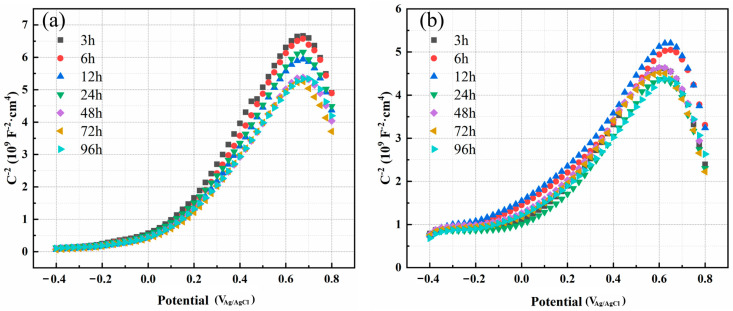
Mott–Schottky plots of passive film formed on C276 alloy (**a**) and 654SMO stainless steel (**b**) at different immersion time.

The Mott–Schottky curves of the passive film formed on the surface of C276 alloy and 654SMO stainless steel after immersion in FGD condensate for different times are shown in Figure 3. At the 0~0.6 V range, M–S curves show a positive slope implying that the passive film displays n-type semiconductor traits. Oxygen vacancies and interstitial cations act as the primary donor carriers within this range [26]. In contrast, at 0.65~0.8 V, M–S curves exhibit a negative slope, signifying a p-type semiconductor. Cation vacancy serves as the main acceptor carrier in this scenario [27]. The *N_D_* and *N_A_* calculated from the slope of the M–S curves are shown in Table 5. The *N_D_* of 654SMO stainless steel under different immersion time is about 11.02~14.23 × 10^20^ cm^−3^, and the *N_D_* of C276 alloy is less than 654SMO, about 7.45~9.82 × 10^20^ cm^−3^. However, the *N_A_* of 654SMO is slightly lower than that of C276 alloy.

**Figure 4:**
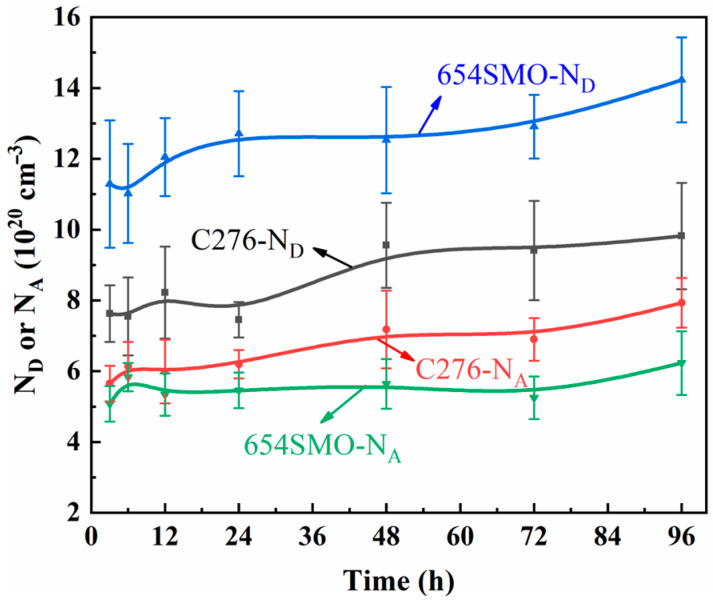
Variation curves of donor and acceptor density (*N_D_*, *N_A_*) with immersion time.

Figure 4 gives the variation curves of N_D_ and N_A_ of the two materials as a function of immersion time. As can be seen, the N_D_ and N_A_ did not change significantly with the initial stage of immersion and started to increase after 24 h immersion. Feng et al. [28] found that the doping concentration of 304 stainless steel decreased gradually with extension of immersion time. Huang et al. [29] believed that long-term immersion at low temperature had little effect on the *N_D_*, while long-term immersion increased the *N_D_* value at high temperatures. The point defect model (PDM) indicates that the change of donor density or acceptor density depends on the generation rate and annihilation rate of carriers [30]. When the rate of generation exceeds the rate of annihilation, point defects will build up in the passive film, leading to a rise in carrier density, and vice versa. This study observes an increase in carrier density in the passive film during extended immersion periods, which might be attributed to the water content serving as a donor carrier, which rises as immersion time progresses.

### 3.2. Electrochemical Behavior of Anodic Polarization at Different Potentials

#### 3.2.1. Potentiostatic Polarization Analysis

**Figure 5:**
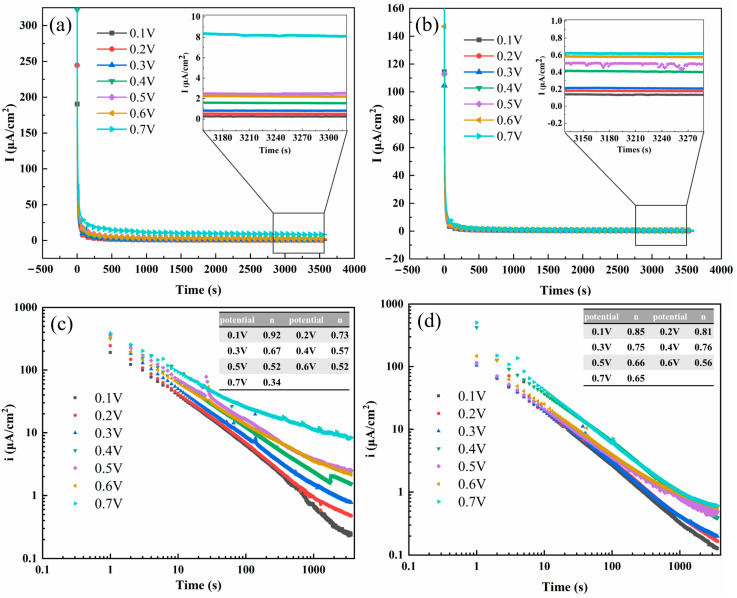
Current–time transients (**a**,**b**) and logi vs. logt transients (**c**,**d**) for C276 alloy (**a**,**c**) and 654SMO stainless steel (**b**,**d**) at different anodic polarization potentials.

**Figure 6:**
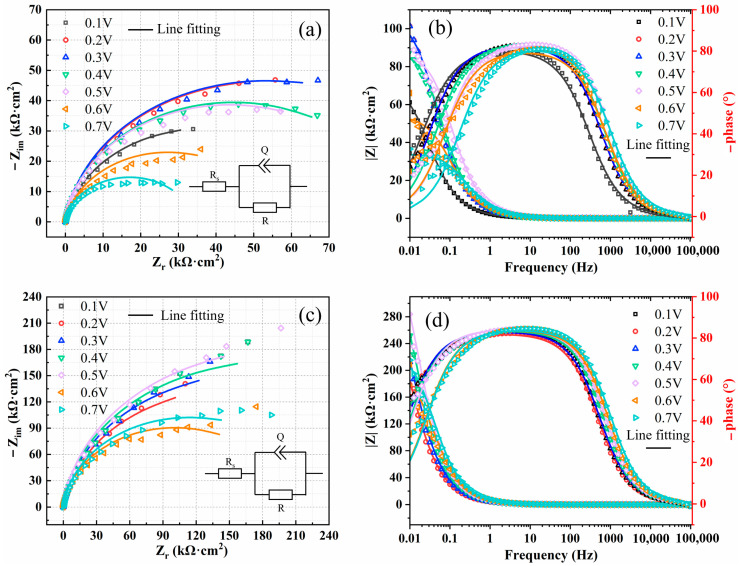
Nyquist (**a**,**c**) and Bode (**b**,**d**) diagrams of C276 alloy (**a**,**b**) and 654SMO stainless steel (**c**,**d**) at different polarization potentials.

Figure 5 shows the current density–time and logi-logt plots for 654SMO stainless steel and the C276 alloy at different polarization potentials. It can be seen from Figure 6a,b that the current density deceases sharply with increase in polarization time and then tends to be stable. The local enlarged view in Figure 5a,b shows that the steady-state current density (*i_ss_*) of the two materials increases with the increase in polarization potentials. Table 6 exhibits the *i_ss_* at different polarization potentials. It is obvious that the *i_ss_* of 654SMO stainless steel at all polarization potentials is lower than that of the C276 alloy, which indicates the dissolution rate of passive film formed on the surface of 654SMO is lower than that of C276.

The literature showed that the change in current density with polarization time can be expressed by the following empirical formula [31,32]:

(4) $$i=A\cdot t^{-n}$$

Taking the logarithm of both sides for the above formula:

(5) logi=logA− n·logt 

where *i* is the current density, *A* is a constant, and *n* is the passivation index. According to Equation (5), the value of *n* can be obtained from the slope of log*i*-log*t* line. Figure 5c,d give the *n* values of the two materials at different potentials. The *n* value indirectly indicates the formation rate of passive film on the fresh metal surface, and its value depends on the applied potential [31,33]. When the value of *n* approaches 1, it results in the formation of a dense passive film with excellent protective properties on the surface. Conversely, when the value of *n* is close to or less than 0.5, a porous passive film develops on the metal surface due to dissolution and precipitation. Analysis of Figure 5c,d reveals that as the polarization potential increases, the value of *n* decreases. The *n* value of C276 alloy at 0.7 V is only 0.3, which deviates from 1 seriously. Except at 0.1 V, the n value of 654SMO is higher than that of C276 alloy.

#### 3.2.2. Electrochemical Impedance Spectroscopy

The EIS results of C276 and 654SMO after polarization at different potentials for 1 h are shown in Figure 6. From observation of Figure 6, the Nyquist curves of the two materials present incomplete capacitance arc, and the arc diameter increases first and then decreases as the potential increases from 0.1 to 0.7 V. Table 7 shows that the oxide film resistance of 654SMO is larger than the C276, and the resistance of the two materials increases first and then decreases with the increase in applied potential.

The effective capacitance (*C*_eff_) extracted from the *CPE* element can help to analyze the electrochemical performance of the passive film [15,17,19]. Orazem et al. summarized three formulas to obtain the *C*_eff_ [34,35,36]:

(6) $$C_{\mathrm{eff}}=Q^{1/n}R_{s}^{\left(1-n\right)/n}$$

(7) $$C_{\mathrm{eff}}=Q^{1/n}R_{f}^{\left(1-n\right)/n}$$

(8) $$C_{\mathrm{eff}}=gQ{\left(\rho_{d}\varepsilon \varepsilon_{0}\right)}^{1-n}$$

In this study, Equation (6) is applied to obtain the *C*_eff_. Table 7 shows the *C*_eff_ values of the two materials at different polarization potentials.

**Figure 7:**
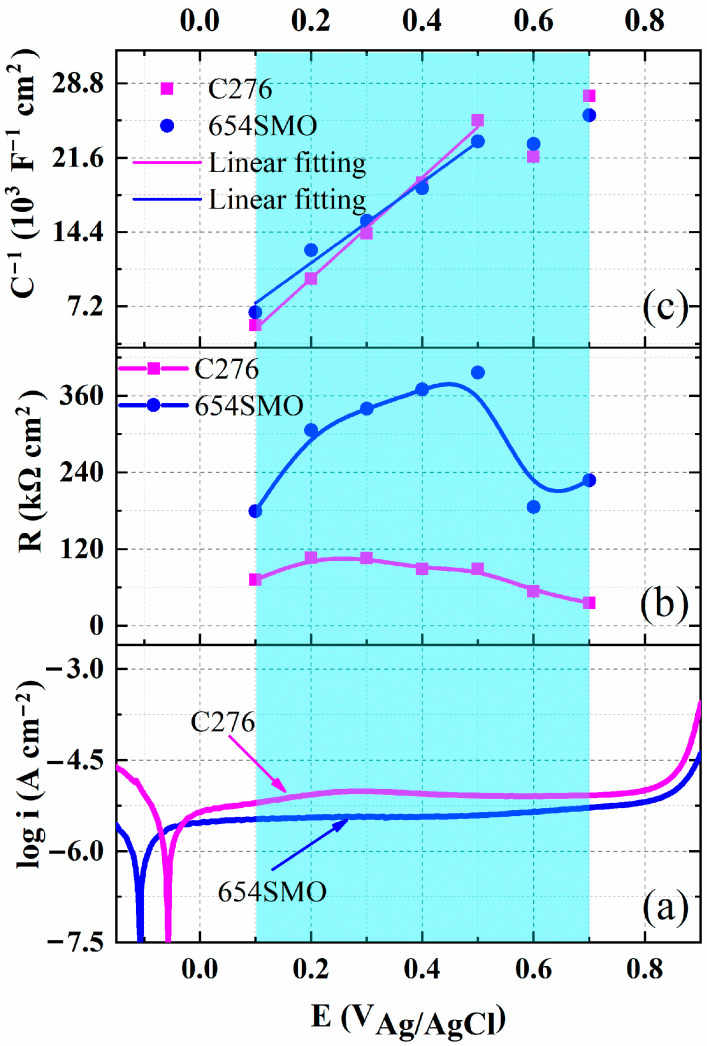
Potentiodynamic polarization curves (**a**) of the two materials, variation in polarization resistance (**b**) and reciprocal of effective capacitance (**c**) with polarization potential. The blue shaded area indicates the range of potential changes between 0.1~0.7 V.

Bojinov et al. [37] believed that there was a relationship between the reciprocal of *C*_eff_ (*C*^−1^) and the applied potential (*E*):

(9) $$\frac{dC^{-1}}{dE}=\frac{1-\alpha }{\varepsilon_{0}\varepsilon E_{0}}$$

where *ε*_0_ is the vacuum dielectric constant (8.8542 × 10^−14^ F/cm), *ε* is the dielectric constant (15.6), *α* is the polarization of the film/solution interface (0.7), and *E*_0_ is the electric field strength. The value of the field strength can be obtained by linear fitting between the reciprocal of capacitance (*C*^−1^) and the potential (*E*). Figure 7 shows variation in polarization resistance and reciprocal of effective capacitance with polarization potential. The polarization resistance of 654SMO is greater than that of the C276 alloy. In the potential range of 0.1~0.4 V, there is a linear relationship between *C*^−1^ and potential, and the field strength E_0_ can be obtained according to the slope of the fitted line.

#### 3.2.3. Mott–Schottky Analysis and Point Defect Diffusivity

**Figure 8:**
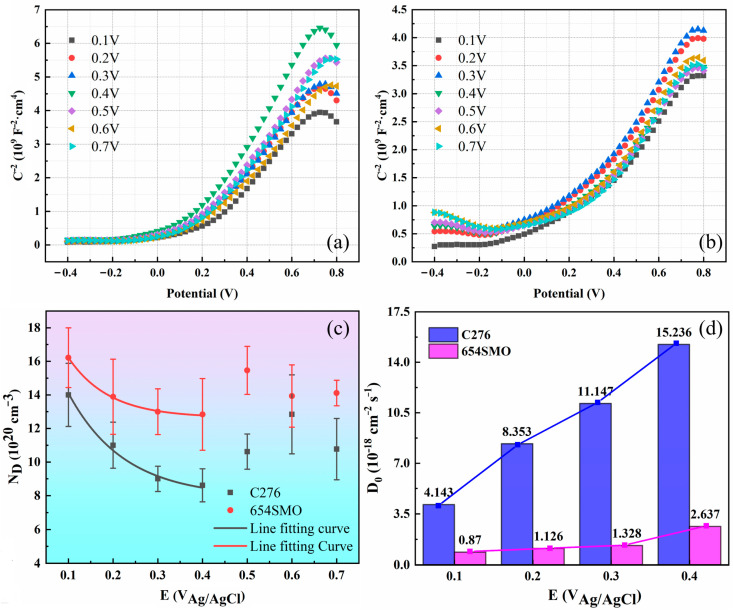
Mott–Schottky curves of C276 (**a**) and 654SMO (**b**) at different potentials, variation in donor density (**c**) and point defect diffusivity (**d**) with polarization potential.

Figure 8a,b show the M–S curves of the two materials at different applied potentials. The positive slope of M–S curves indicates the passive film formed by anodic polarization mainly exhibits n-type semiconductor characteristics. The donor density of the two materials is obtained from Equation (2), as shown in Figure 8c. Sikora et al. [38] established the relationship between donor density and polarization potential:

(10) $$N_{D}=\omega_{1}\exp\left(-bE_{ff}\right)+\omega_{2}$$

The values of each parameter in Equation (10) can be obtained by nonlinear fitting of Figure 8c.

Point defect diffusivity (*D*_0_) is a significant parameter to describe point defect transport and passive film performance [39]. There is a relationship between the *D*_0_ and *ω*_2_ in Equation (10) [39]:

(11) $$\omega_{2}=\frac{-J_{0}}{2KD_{0}}$$

Then, *D*_0_ can be denoted as follows [38]:

(12) $$D_{0}=\frac{-J_{0}}{2K\omega_{2}}=\frac{-J_{0}RT}{2F\omega_{2}E_{0}}$$

where *J*_0_ is the steady flux of door density of passive film, which has the following relationship with the steady current density (*i_ss_*):

(13) $$J_{0}=\frac{{-i}_{ss}}{2e}$$

Thus, *D*_0_ can be expressed as follows:

(14) $$D_{0}=\frac{i_{ss}RT}{4eF\omega_{2}E_{0}}$$

where *R* is gas constant (8.314 J·mol^−1^·K^−1^), *F* is Faraday constant (96,596 C·mol^−1^), *E*_0_ can be obtained from Equation (9), and *ω*_2_ can be obtained from Equation (10). Thus, the point defect diffusivity can be obtained, as shown in Figure 8d. From Figure 8d, the *D*_0_ value increases with the increase in polarization potential, and *D*_0_ of 654SMO is apparently lower than that of C276 alloy, which demonstrates that 654SMO exhibits higher corrosion resistance in FGD condensates.

### 3.3. Composition and Thickness Analysis of Passive Film

#### 3.3.1. Surface Analysis of the Passive Film

**Figure 9:**
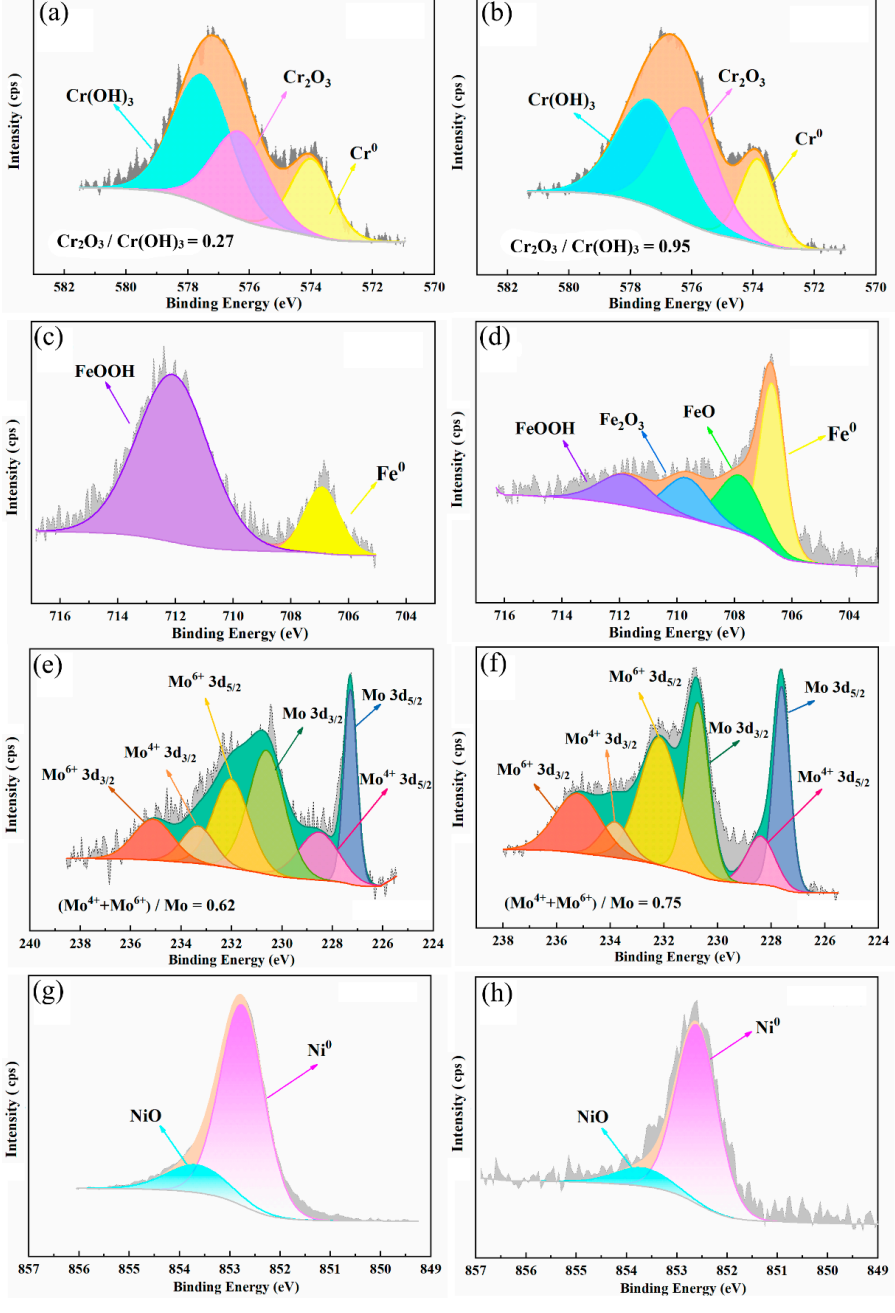
Detailed XPS spectra of Cr 2p_3/2_ (**a**,**b**), Fe 2p_3/2_ (**c**,**d**), Mo 3d (**e**,**f**), and Ni 2p_3/2_ (**g**,**h**) of the passive film formed on C276 alloy (**a**,**c**,**e**,**g**) and 654SMO stainless steel (**b**,**d**,**f**,**h**).

**Figure 10:**
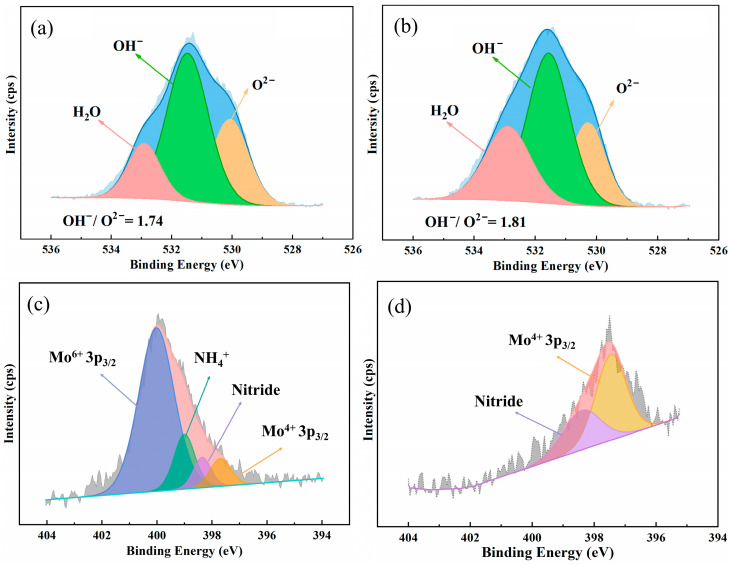
Detailed XPS spectra of O 1 s of the passive film formed on C276 (**a**) and 654SMO (**b**), N 1 s spectra of 654SMO after 0 s (**c**) and 10 s (**d**) etching.

Figure 9 and Figure 10 display the detailed spectra of Cr 2p_3/2_, Fe 2p_3/2_, Mo 3d, Ni 2p_3/2_, O 1s and N 1s after deconvolution. The binding energy and full width at half maximum (FWHM) of each component can be found in Table 8. As seen in Figure 10, Cr 2p_3/2_ spectra of 654SMO and C276 show three components: metallic chromium (Cr^0^), chromium oxide (Cr_2_O_3_), and chromium hydroxide (Cr(OH)_3_). In the case of 654SMO, there are four chemical variations of Fe, including metallic Fe, FeO, Fe_2_O_3_, and FeOOH. However, for C276, only two components are identified in the Fe 2p3/2 spectra, specifically metallic Fe and FeOOH, potentially due to the lower Fe content in the C276 alloy.

Mo 3d integral peaks are split into three Mo 3d_3/2_-Mo3d_5/2_ doublets. The metallic state along with four-valence (Mo^4+^) and six-valence (Mo^6+^) species are observed in the Mo spectra. Ni 2p_3/2_ spectra can be divided into a strong metallic Ni peak and a weak NiO peak. O 1s spectra can be separated into three peaks, namely, O^2−^, OH^−^, and H_2_O. Figure 9 and Figure 10 also give the ratio of Cr_2_O_3_/Cr(OH)_3_, (Mo^4+^ + Mo^6+^)/Mo, and O^2−^/OH^−^. It is found that the ratio of Cr_2_O_3_/Cr(OH)_3_ and O^2−^/OH^−^ of 654SMO is higher than that of C276. The ratio of (Mo^4+^ + Mo^6+^)/Mo of 654SMO is lower than that of C276.

For 654SMO stainless steel, the N 1s spectrum was observed in the passive film. Due to the overlaps of the N 1s peaks with the Mo 3p peaks, it is necessary to analyze the Mo 3p spectrum simultaneously when analyzing the N 1s spectrum, as shown in Figure 10. Without sputtering, there are two peaks related to N, namely, ${\mathrm{NH}}_{4}^{+}$ and nitride. After 10s of sputtering, only nitride remains. In the acidic solution, the enrichment of ${\mathrm{NH}}_{4}^{+}$ on the surface of the passive film helps to increase the pH value in the pitting area and form re-passivation effect, delaying the rupture of the passive film and the initiation of the pitting corrosion [40,41,42].

#### 3.3.2. Depth Profiles of the Passive Film

**Figure 11:**
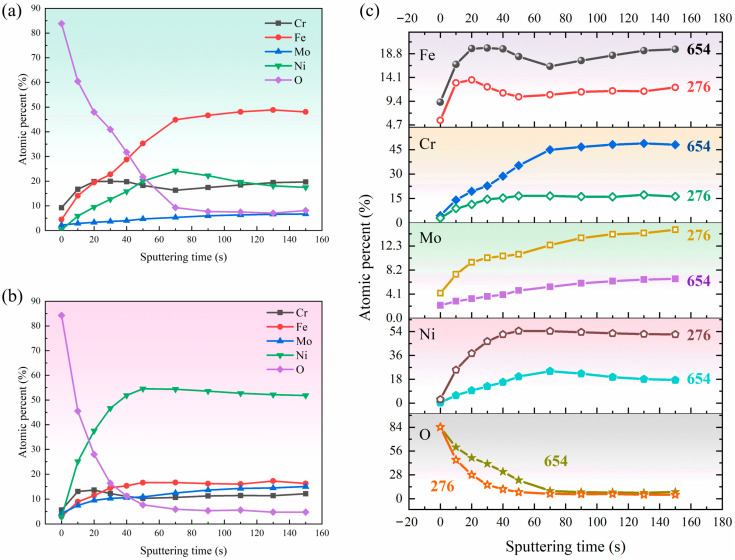
XPS depth profiles of the elements in the passive film of 654SMO (**a**) and C276 (**b**) after immersion in the simulated condensate for 24 h and comparison of element distribution for the two materials (**c**).

Figure 11a,b display the atomic percentage of Cr, Fe, Mo, Ni, and O element in the passive film following sputtering for varying durations. The progressive decline in oxygen content, accompanied by an increase in iron and nickel levels as sputtering time increases, indicates the transition from the metal surface to the substrate. Figure 11c shows the difference in composition distribution of passive film formed on 654SMO and C276. An analysis reveals that 654SMO alloy exhibits a notably higher atomic percent of Cr and Fe compared to C276, whereas the atomic percent of Mo and Ni in 654SMo is lower than C276.

**Figure 12:**
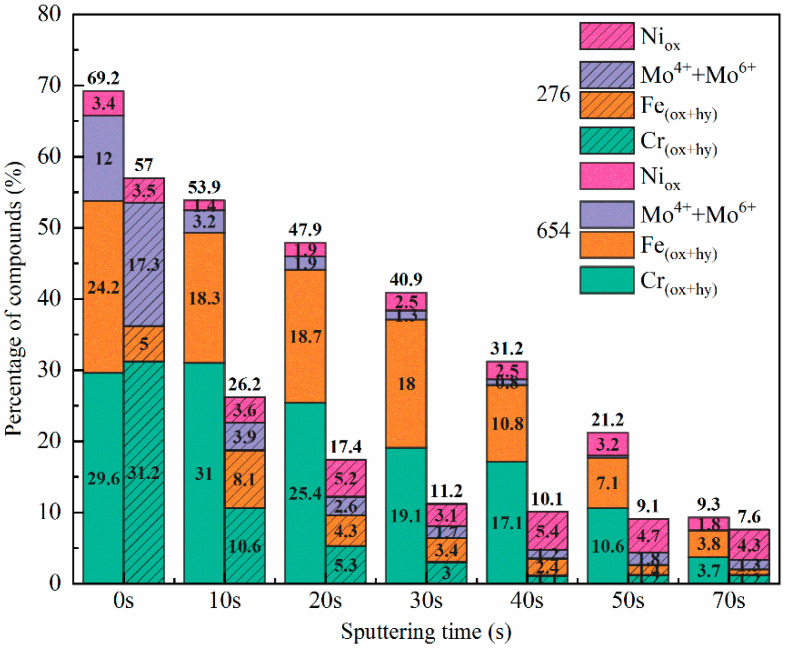
Variation in passive film components of 654SMO and C276 with sputtering time.

Figure 12 shows the fraction of various compounds in the passive film formed on the 654SMO and C276 under different sputtering times. It is evident that the predominant constituents of the passive films on both materials are Cr, Fe oxides, and hydroxides, while Mo and Ni compounds represent a smaller fraction. The overall compound concentration in the passive layer of 654SMO consistently surpasses that of C276 across all sputtering intervals. Following 50 s of sputtering, the compound content in the passive film of C276 falls below 10%, whereas the passive film of 654SMO reaches this threshold only after 70 s of sputtering. This observation suggests that 654SMO forms a more substantial passive film in the FGD solution. Of course, the thickness of the passive film is not the only criterion for high or low corrosion resistance [43]. When combined with the findings presented in Figure 8, it can be observed that the lower diffusion coefficient of defects within the passive film of 654SMO suggests that a more substantial and compact passive film is generated in the simulated environment.

From the results in Figure 4 and Figure 8, the donor density of 654SMO is higher than that of C276, while the acceptor density is lower than that of C276. This phenomenon can be explained from the constituent of the passive film. The semiconductor characteristics of passive film developed on the stainless steel or alloy surface rely on the film composition and structure. For example, Cr_2_O_3_, FeCr_2_O_4_, MoO_2_, and NiO exhibit p-type semiconductor behavior [43,44], while Fe oxides, especially Fe_2_O_3_, FeOOH, and MoO_3_ exhibit n-type semiconductor behavior [43,44]. The performance of the passive film in semiconductors relies on the involvement of individual oxides and the presence of cation or anion vacancies in these oxides. The increased donor density in the passive film of 654SMO could be ascribed to the abundant Fe oxide content. Conversely, the reduced acceptor density in 654SMO suggests the Cr oxide layer with minimal defect density. The low content of Ni oxide in the passive film of the two materials may be related to the high Gibbs free energy of formation [45] (ΔG_f, Cr2O3_ = −1058 kJ/mol, ΔG_f, Cr(OH)3_ = −1064 kJ/mol, ΔG_f, FeCr2O4_ = −1344 kJ/mol, ΔG_f, Fe3O4_ = −1118 kJ/mol, ΔG_f, Fe2O3_ = −742 kJ/mol, ΔG_f, NiO_ = −212 kJ/mol).

In conclusion, the results of electrochemical experiments show that 654SMO has better corrosion resistance than C276 in the FGD condensate, which is attributed to the thicker and denser passive layer of 654SMO stainless steel.

## 4. Conclusions

The electrochemical behavior and surface film chemistry of 654SMO super austenitic stainless steel and the C276 nickel-based alloy after natural immersion and anodic polarization in flue gas desulfurization condensate at 80 °C were investigated, and the main conclusions obtained were as follows:

(1) The open circuit potential of 654SMO is higher than that of C276. The results of potentiodynamic polarization curves show that the transpassive potential of 654SMO is higher than that of C276, and the passive current density of 654SMO is lower than that of C276.
(2) With the prolongation of immersion time, the electrochemical impedance of the two materials increases first and then decreases. The impendence of 654SMO under different immersion time is greater than C276. The passive film exhibits n-type semiconductor characteristics at 0~0.6 V and p-type at 0.65~0.8 V. The donor density of 654SMO is higher than that of C276, while the acceptor density is lower than that of C276.
(3) The steady current density of 654SMO and C276 increases with the increase in polarization potential from 0.1 V to 0.7 V. With the increase in polarization potential, the impedance of the two materials increases first and then decreases. 654SMO stainless steel has a significantly smaller diffusion coefficient of point defects than C276 alloy.
(4) With increasing sputtering time, the atomic percentage of Cr, Fe, and Ni increases, and the atomic percentage of O decreases for both materials. The total oxide content of passive film formed in 654SMO is higher than that in C276 at different sputtering times.

## Figures and Tables

**Table 1:** Chemical compositions of 654SMO stainless steel and C276 alloy in wt.% (PREN = Cr% + 3.3 × (Mo + 0.5W)% + 16 × N%).

Material	Fe	Ni	Cr	Mo	Cu	Mn	Co	Si	N	W	PREN
654SMO	42.25	22.03	24.56	7.02	0.33	3.19	-	0.071	0.509	-	56
C276	5.61	57.57	15.76	15.57	-	0.53	1.53	0.04	-	3.38	73

**Table 2:** Chemical composition of the simulated desulfurized flue gas condensates.

Agents	H_2_SO_4_ (mL)	HNO_3_ (mL)	HCl (mL)	Na_2_SO_4_ (g)	H_2_O (L)	pH
Content	10	9	3	1.88	4	1.1

**Table 3:** Electrochemical parameters obtained from the potentiodynamic polarization curves.

Materials	E_corr_ (V_Ag/AgCl_)	i_p_ (μA·cm^−2^)	E_tr_ (V_Ag/AgCl_)
654SMO	−0.123 ± 0.015	4.12 ± 0.35	0.917 ± 0.002
C276	−0.067 ± 0.014	8.30 ± 0.58	0.891 ± 0.017

**Table 4:** Fitted electrochemical parameters for EIS of C276 alloy and 654SMO stainless steel in the simulated flue gas condensate.

		Rs (Ω·cm^2^)	Q_1_ 10^−6^ (Ω·cm^−2^·s^n^)	n_1_	R_1_ (kΩ·cm^2^)	Q_2_ 10^−6^ (Ω·cm^−2^·s^n^)	n_2_	R_2_ (kΩ·cm^2^)	χ^2^ (10^−4^)
276	3 h	13.49	75.68	0.92	1.47	40.35	0.81	63.66	1.57
	6 h	13.01	85.77	0.91	1.79	36.84	0.83	67.58	1.48
	12 h	12.83	75.64	0.92	2.28	31.65	0.82	85.51	2.52
	24 h	13.13	72.78	0.90	2.03	17.76	0.70	205.4	0.77
	48 h	12.74	65.28	0.91	2.11	14.35	0.77	174.6	0.99
	72 h	13.40	72.95	0.91	8.52	19.08	0.68	152.3	2.24
	96 h	13.27	55.66	0.93	3.03	24.03	0.71	87.14	9.33
654	3 h	12.67	43.94	0.91	65.31	16.73	0.75	3830	1.04
	6 h	12.84	50.31	0.91	228.7	19.14	0.81	4374	5.51
	12 h	12.04	73.94	0.89	431.2	41.35	0.83	5135	3.80
	24 h	12.98	42.98	0.92	102.4	10.65	0.68	6358	5.04
	48 h	12.46	38.88	0.91	305.4	20.97	0.94	7784	1.59
	72 h	13.17	47.06	0.91	245.7	18.05	0.81	6183	7.85
	96 h	13.27	50.84	0.91	254.1	22.74	0.88	4753	1.39

**Table 5:** Donor and acceptor density (*N_D_*, *N_A_*) of the passive film formed on C276 and 654SMO at different immersion times.

Time	C276		654SMO	
	*N_D_* (10^20^ cm^−3^)	*N_A_* (10^20^ cm^−3^)	*N_D_* (10^20^ cm^−3^)	*N_A_* (10^20^ cm^−3^)
3 h	7.63	5.65	11.29	5.08
6 h	7.55	6.13	11.02	5.83
12 h	8.22	5.99	12.05	5.34
24 h	7.45	6.20	12.71	5.46
48 h	9.56	7.18	12.53	5.64
72 h	9.41	6.90	12.91	5.25
96 h	9.82	7.93	14.23	6.23

**Table 6:** Steady-state current density (μA/cm^2^) of the two materials polarized at different potentials.

	0.1 V	0.2 V	0.3 V	0.4 V	0.5 V	0.6 V	0.7 V
C276	0.307 ± 0.112	0.619 ± 0.114	0.826 ± 0.048	1.129 ± 0.382	1.644 ± 0.784	1.588 ± 0.5316	5.490 ± 1.3440
654SMO	0.129 ± 0.016	0.167 ± 0.011	0.197 ± 0.009	0.391 ± 0.028	0.495 ± 0.017	0.569 ± 0.151	0.641 ± 0.134

**Table 7:** Fitted electrochemical parameters for EIS of C276 alloy and 654SMO stainless steel in the simulated flue gas condensate.

Material	Potential (V)	Rs (Ω·cm^2^)	Q10^−6^ (Ω·cm^−2^·s^n^)	n	R (kΩ·cm^2^)	*C*_eff_ (μF·cm^−2^)	χ^2^ (10^−3^)
C276	0.1	13.21	87.22	0.8991	71.96	192.3933	1.55
	0.2	13.52	54.54	0.9161	106.3	99.8503	1.63
	0.3	12.44	41.88	0.9224	105.9	70.8874	1.61
	0.4	11.83	35.22	0.9263	89.18	56.9153	1.66
	0.5	13.13	23.09	0.9269	88.87	36.2362	5.05
	0.6	13.46	36.76	0.8968	54.37	75.0651	2.58
	0.7	12.5	27.3	0.9097	35.91	48.7085	4.50
654SMO	0.1	12.25	68.69	0.8921	179.5	155.1235	1.74
	0.2	13.01	49.71	0.9151	305.9	90.6178	1.34
	0.3	12.66	42.47	0.9258	339.8	70.2950	1.37
	0.4	12.51	33.97	0.9312	369.5	53.1235	1.80
	0.5	12.39	29.53	0.9365	396.3	44.0628	1.72
	0.6	12.04	26.68	0.9367	185.8	39.4085	3.37
	0.7	11.87	23.07	0.9357	227.6	33.9273	3.42

**Table 8:** Binding energy and full width at half maximum (FWHM) of XPS peaks used for deconvolution.

Constituents	654SMO		C276	
	Binding Energy (eV)	FWHM (eV)	Binding Energy (eV)	FWHM (eV)
Fe	706.7	1.05	706.9	1.5
FeO	707.9	1.86	-	-
Fe_2_O_3_	709.7	1.86	-	-
FeOOH	711.9	2.25	712.1	3.06
Cr	573.8	1.41	573.9	1.77
Cr_2_O_3_	576.1	2.28	576.3	2.09
Cr(OH)_3_	577.3	2.61	577.5	2.13
Ni	852.6	1	852.7	1.07
NiO	853.6	1.34	853.6	0.85
Mo 3d_5/2_	227.3	0.63	227.6	0.75
Mo^4+^ 3d_5/2_	228.5	1.96	228.4	1.3
Mo 3d_3/2_	230.6	1.75	230.7	1.03
Mo^6+^ 3d_5/2_	232.1	1.76	232.2	1.84
Mo^4+^ 3d_3/2_	233.3	1.58	233.8	1.31
Mo^6+^ 3d_3/2_	235.1	1.92	235.2	2.09
O^2−^	530.1	0.58	530.3	1.39
OH^−^	531.5	1	531.6	1.68
H_2_O	532.9	0.37	532.8	1.82
Mo^6+^ 3p_3/2_	400.0	1.58		
Mo^4+^ 3p_3/2_	397.7	0.92		
Nitride	398.4	0.78	-	-
${\mathrm{NH}}_{4}^{+}$	399.0	0.93	-	-

## Data Availability

Data is unavailable due to privacy or ethical restrictions.
